# Hypothetical protein Cpn0423 triggers NOD2 activation and contributes to *Chlamydia pneumoniae-*mediated inflammation

**DOI:** 10.1186/s12866-017-1062-y

**Published:** 2017-07-11

**Authors:** Hong-liang Chen, Guo-zhi Dai, An-wen Zhou, Ran-hui Li, Hong-xia Yuan, Jing Xiang, Xiao-xing You, Ou Ran, Yi-mou Wu

**Affiliations:** 1 Department of Clinical Laboratory, ChenZhou NO.1 People’s Hospital, ChenZhou, 423000 China; 2 Institute of Translational Medicine, University of South China, ChenZhou, 423000 China; 3 Institute of Pathogenic Biology, Medical College, University of South China, Hengyang, 421001 China

**Keywords:** *Chlamydia pneumoniae*, Cpn0423, NOD2, Inflammatory

## Abstract

**Background:**

*Chlamydia pneumoniae* (*C. pneumoniae)* is pathogenic to humans, by causing pulmonary inflammation or bronchitis in both adolescents and young adults. However, the molecular signals linking *C. pneumoniae* components to inflammation remain elusive. This study was to investigate the effect of Chlamydia-specific Cpn0423 of *C. pneumoniae* on *C. pneumoniae*-mediated inflammation.

**Results:**

Cpn0423 was detected outside of *C. pneumoniae* inclusions, which induced production of several cytokines including macrophage inflammatory protein-2 (MIP-2) and interleukins (ILs). Production of the Cpn0423-induced cytokines was markedly reduced in cells pretreated with NOD2-siRNA, but not with negative control oligonucleotides. Mice treated with Cpn0423 through intranasal administration exhibited pulmonary inflammation as evidenced by infiltration of inflammatory cells, increased inflammatory scores in the lung histology, recruitment of neutrophils and increased cytokines levels in the BALF.

**Conclusion:**

Cpn0423 could be sensed by NOD2, which was identified as an essential element in a pathway contributing to the development of *C. pneumoniae* -mediated inflammation.

## Background

*Chlamydia pneumoniae* (Cpn), a common non-viral intracellular pathogen, is known as a leading cause of human respiratory tract infections worldwide, and responsible for up to 10% of community acquired pneumonias [1]. *C. pneumoniae* mainly infects the mucosal surfaces of human respiratory tract, causing various respiratory diseases, such as pneumonia, bronchitis and pharyngitis [2, 3]. It is also associated with the development of or exacerbation of chronic obstructive pulmonary disorder [4]. *C. pneumoniae* undergoes a unique biphasic developmental form, including the non-infectious metabolically active reticulate body (RB) and the infectious elementary body (EB) [5]. Upon internalization of *C. pneumoniae* by a host cell, it is believed to trigger immune responses and induce chronic inflammation with the release of cytokines such as IL-6, IL-10 and TNF-alpha [6, 7]. Due to this link with important chronic inflammatory diseases, and the fact that *C. pneumoniae* infection occurs worldwide with a seroprevalence of 70% in the adult population [8, 9], understanding the mechanisms underlying inflammatory response during *C. pneumoniae* infection is of great importance to dissect its potential role in these chronic inflammatory diseases.

*C. pneumoniae* differs from most bacteria in that it lacks the ability to produce its own adenosine triphosphate (ATP). Thus, *C. pneumoniae* has evolved the ability to secrete proteins into both the inclusion membrane and host cell cytoplasm, which may help the organisms of *C. pneumoniae* to take up nutrients and energy from host cells, and to maintain the integrity of host cells by preventing the infected host cells from host immune response [10, 11]. Cpn0423 is a Chlamydia-specific hypothetical protein which is highly conserved among all chlamydial genomes [12], suggesting an important role of Cpn0423 in maintaining *C. pneumoniae* intracellular infection.

Innate immune system represents the first line of defense against foreign pathogens, such as bacteria, viruses and fungi [13, 14]. The two principal families of pathogen detectors are the Toll-like receptors (TLRs) and the nucleotide-binding oligomerization domain-like receptors (NLRs) [15]. TLRs are embedded in the plasma membrane and recognize conserved pathogen-associated molecules. Studies from our laboratory indicated that heat shock protein 10 (HSP10) of *C. pneumoniae* could elicit inflammatory reactions mediated by TLR4 [16]. Others also confirmed that both TLR2 and TLR4 signaling induce early cytokine and chemokine production during *C. pneumoniae* respiratory infection [17]. Surprisingly, the IFN-γ could be induced by *C. pneumoniae* in the absence of TLR4/MyD88 signaling pathways [18], suggesting a potential contribution of intracellular recognition receptors like NLRs to *C. pneumoniae*–induced inflammatory cytokines production. NLRs, also called caspase recruitment domain-containing proteins, are a group of evolutionarily conserved intracellular pattern recognition receptors (PRRs) that play a vital role in innate immune responses against certain bacterial infections [15, 19]. Nucleotide-binding oligomerization domain-containing protein 2 (NOD2) is a large multi-domain protein, that serves as a general sensor for both intracellular Gram-positive and Gram-negative bacteria and that mediates responsiveness to molecules in the cytoplasm originated from bacteria, including muramyl dipeptide conserved in peptidoglycans [20]. NOD2 generally prevails in the cytoplasm in a dormant form. Once activated, it will elicit specific immune responses by the recruitment of specific adaptor molecules as well as effector molecules like inflammatory procaspases and kinases [21]. Although NOD2 was reported to be involved in the activation of innate immune defenses against intracellular *C. pneumoniae* [6], further details of the molecular signals linking NOD2 to *C. pneumoniae* infection remains largely unknown.

Our previous studies already identified that Cpn0423 protein was expressed and located in *C. pneumoniae* infected host cell cytosol. Since NOD2 was also one of the cytosolic PRRs, we hypothesized that Cpn0423 is a potent activator that stimulates specific inflammatory responses via a NOD2-dependent mechanism. We treated bone marrow-derived macrophages (BMDMs) with Cpn0423 and measured cytokine responses. We assessed the specific roles of NOD2 signaling by using NOD2-siRNA oligonucleotides. We demonstrated the pro-inflammatory properties of Cpn0423 in a mouse model in vivo*.* Finally, we found that Cpn0423 acts as a signal for NOD2 receptor, which provides important information on the mechanism of *C. pneumoniae* pathogenesis.

## Methods

### Organism and *C. pneumoniae* infection

*C. pneumoniae* strain AR39 was kindly provided by Dr. Zhong (University of Texas, San Antonio, TX). *C. pneumoniae* was propagated, purified and quantified as previously described [22]. For infection, HEp-2 cells were determined to be free of Mycoplasma contamination, and inoculated with *C. pneumoniae* AR39 organisms at an MOI of 0.5 in the presence of 2 μg/ml of cycloheximide, and grown in RPMI 1640 medium supplemented with 10% FBS at 37 °C in a 5% CO2 environment for 72 h as indicated in the following experiments.

### *C. pneumoniae* gene cloning and fusion protein expression

Gene coding for the ORF Cpn0423 was amplified from *C. pneumoniae* AR-39 genomic DNA and cloned into pGEX vectors (TaKaRa, Dalian, China). The special primers for full-length Cpn0423 covering residues (condon 1-429) were as follows**:** forward primer 5′-CGC-GGATCC (BamH I) –ATGTTGGATAATGAATGGAA AGC-3′, backward primer 5′-TTTTCCTTTT-GCGGCCGC (Not I) -TTAACGAACTAA CGCAGCATTT-3′; The gene Cpn0423 was expressed as a fusion proteins with GST fused to the N-terminus of the chlamydial proteins as previously described [23]. Overexpression of the fusion protein was induced by 0.2 mM Isopropyl β-D-Thiogalactoside (IPTG) (TaKaRa). The recombinant fusion protein was extracted by lysing the *E. coli* BL21 (DE3) (Novagen) transformed with pGEX-6P-Cpn0423 recombinant plasmid via sonication in a Triton-X100 lysis buffer (1% Triton X-100, 1 mM phenylmethanesulfonyl fluoride, 75 U/ml aprotinin). After a high-speed centrifugation to remove debris, the fusion protein was further purified by affinity chromatography using Glutathione Sepharose 4B from ameshame pharmacia (Zkcy, BeiJing, China) and was analyzed by SDS-polyacrylamide gel electrophoresis (SDS-PAGE) and Coomassie Blue staining. Pierce High-Capacity Endotoxin Removal Resin (BXGK, BeiJing, China) was used to remove endotoxin from purified Cpn0423 fusion protein following its protocol. Final endotoxin concentration of purified Cpn0423 fusion protein used in this study was less than 60 pg/mL, determined by Limulus amebocyte lysate kit (BXGK) based on a standard curve. The endotoxin-removed Cpn0423 fusion protein was used to immunize mice for producing polyclonal antibody (pAb) as previously described [24]. Briefly, mice were immunized three times intramuscularly with 100 μg fusion protein and incomplete Freund’s adjuvant (IFA) and three times intravenously with 100 μg fusion protein without IFA.

### Immunofluorescence assay

The immunofluorescence assay was carried out as described previously [22, 25]. Briefly, Hep-2 cells, with *C. pneumoniae* infection grown on coverslips were fixed with 2% paraformaldehyde (Sigma) for 30 min at room temperature (RT), followed by permeabilization with 1% saponin (Sigma) for an additional 30 min. After washing and blocking, the cell samples were subjected to antibody and chemical staining. Hoechst (Sangon Biotech, Shanghai, China) was used to visualize DNA. A rabbit anti-*C. pneumoniae* organism antibody (raised with *C. pneumoniae* AR39 organisms, unpublished data) and a goat anti-rabbit IgG secondary antibody conjugated with Cy2 (green; Sangon Biotech) were used to visualize *C. pneumoniae* AR39 organisms. A mouse anti-Cpn0423 antibody (raised with the Cpn0423 fusion protein, unpublished data) and a goat anti-mouse IgG conjugated with Cy3 (red; Sangon Biotech) were used to visualize Cpn0423 antigen. Uninfected Hep-2 cells were stained for control. In addition, for antibody absorption experiments, the primary antibodies were further absorbed with either the corresponding or heterologous fusion proteins CPAF [26] immobilized onto glutathione-conjugated agarose beads prior to staining, which was used to prove the antibody binding specificities. The absorption was carried out by incubating the antibodies with bead-immobilized antigens for 1 h at room temperature followed by pelleting the beads. The remaining supernatants were used for immunostaining. The immunofluorescence images were acquired using an ECLIPSE TE2000-5 fluorescence microscope (Nikon, Inc., Japan) connected to a Sony 3CCD color video camera.

### Cell isolation and culture

Mouse bone marrow derived macrophages (BMDMs) were prepared as described previously with minor modifications [27]. Briefly, bone marrow cells were harvested from the tibias and femurs of mice, re-suspended in RPMI 1640 medium supplemented with 10 ng/ml macrophage colony-stimulating factor (TaKaRa), 10% FBS (Invitrogen), 5% horse serum (Invitrogen) and 1% penicillin/streptomycin (Sigma), seeded in a 96-well plate (2 × 10^5^ cells/well) or 6-well plate (2 × 10^6^ cells/well) and incubated in CO_2_ incubator at 37 °C. Three days later, culture media were changed and the macrophages were ready for experiments at day 5.

### Treatments of cell cultures

Cultured BMDMs were washed twice, and then transferred to a pre-warmed serum-free RPMI 1640 medium containing 0.05% BSA for 1 h before treatment. Cpn0423 was complexed with Pro-Ject™ Protein Transfection Reagent (PTR) (Invitrogen) following the manufacture’s instruction and was incubated for 5 min at room temperature before applied to cell cultures. The cell cultures were then treated in RPMI 1640 supplemented with 10% FBS. For additional control experiments, Cpn0423 or LPS was used in combination with 50 μg/ml Polymyxin B (PMB), as a LPS neutralizer [28] for 10 min before mixed with Pro-Ject™ Reagent. For RNA interference experiments, small interfering RNA against NOD2 (NOD2-siRNA) and small interfering RNA negative control for NOD2 (NOD2-Con-siRNA) were transfected into BMDMs with Lipofectamine 3000 (Invitrogen), incubated at 37 °C for 48 h before applied to cell cultures. NOD2-siRNA and NOD2-Con-siRNA oligonucleotides sequence were as follows: Sense 5′-CGGUGAAAGCGAAUGGAUU-3′, Antisense 5′-AAUCCAUUCGCUUUCAC CG-3′, Control (1) 5′-CGTGAGAGGGGGGACACUU-3′, Control (2) 5′-AAATCAC TTACTGGCGCTCG-3′.

### Cytokine protein measurement

Culture medium and bronchoalveolar lavage fluid (BALF) were prepared and stored at −80 °*C. ELISA* kits (R&D systems, Minneapolis, MN) were used to measure the levels of MIP-2, IL-6 and TNF-α, while the final cytokine concentrations were calculated based on a standard curve constructed in each experiment.

### Quantitative RT-PCR

Quantitative RT-PCR was performed as described previously [27]. After total RNA isolation and cDNA Synthesis, real-time RT-PCR was performed by using the Power SYBR Green Master Mix (Applied Biosystems, CA) according to the manufacturer’s protocols. For RT-PCR experiments, 1 μg of total RNA was reverse-transcribed using SuperScript III reverse transcriptase (Invitrogen) prior to PCR amplification. The sequences of the oligonucleotide primers utilized for real time RT-PCR were as follows: for NOD2, forward primer 5′-CAAACCCTCAGGATAGGAAATGTAG-3′, backward primer 5′- TAAGTGAAGAGTCAGGTGATGGATG-3′; for TLR2, forward primer 5′-GGTTTCTGAGCCAGTACGAGTGTG-3′, backward primer 5′- GGTAGTTCCCTGACGTGCTGTAGA-3′; for GAPDH, forward primer 5′-AACTTTGGCATTGTGGAAGG-3′, backward primer 5′- GGATGCAGGGATGATGTTCT-3′. Final quantifications were calculated by the 2^(−△△Ct)^ method, and GAPDH was selected as a normalizing gene empirically.

### Western blotting

**Fig. 1:**
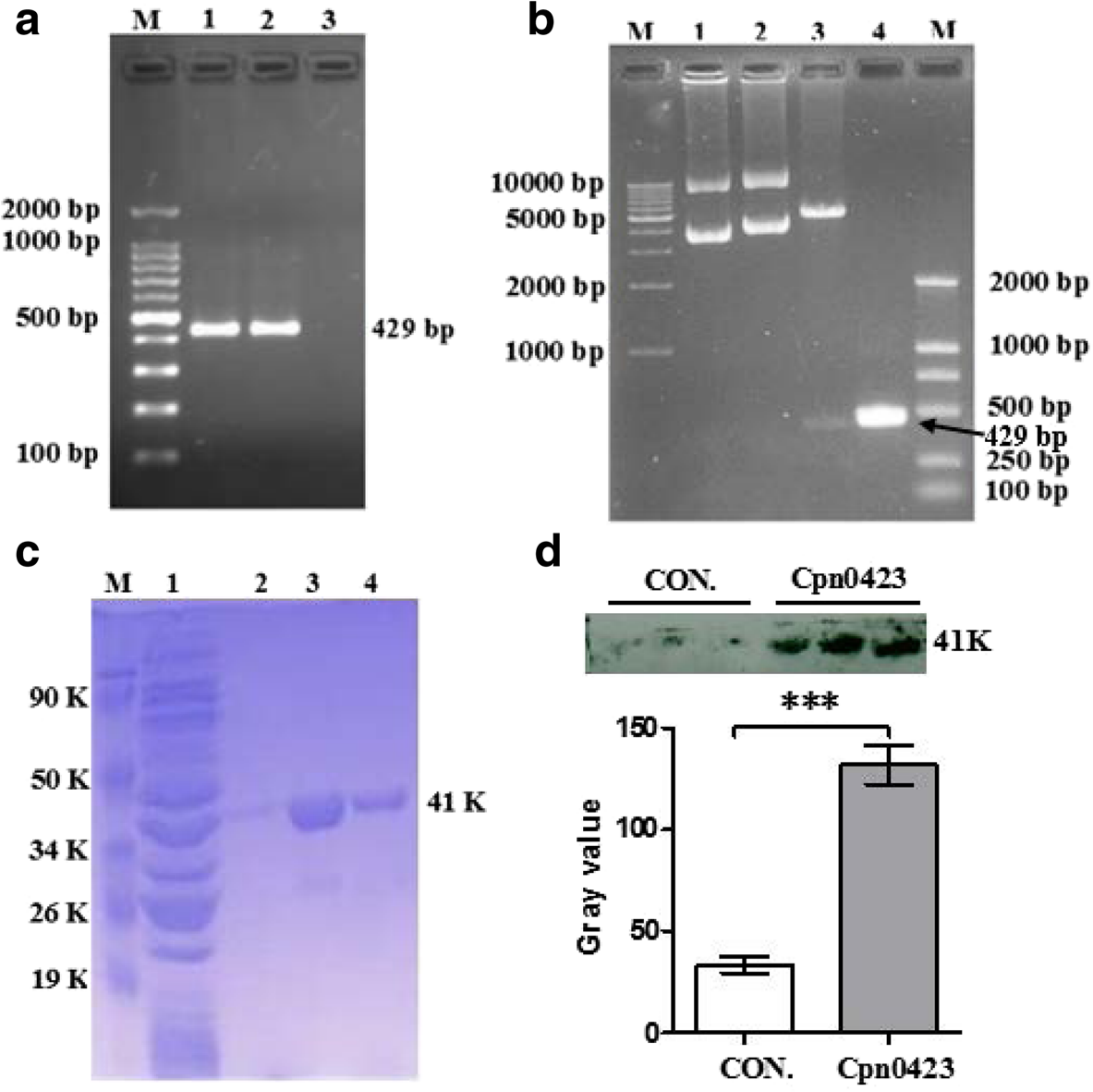
Cloning, expression and purification of Cpn0423 of *C. pneumoniae*. **a** PCR products of Cpn0423 amplified from the *C. pneumoniae* strain AR-39. Lane M, DNA Marker; lanes 1 and 2, PCR amplified products of Cpn0423; lane 3, negative control. **b** Identification and restriction end nuclease analysis of the vector pGEX6p-2/Cpn0423. Lane M, DNA Marker; lane 1, pGEX6p-2 vector; lane 2, pGEX6p-2/Cpn0423 vector; lane 3, pGEX6p-2/Cpn0423 digested with Bam H I and Not I; lane 4, PCR amplified products of Cpn0423. **c** SDS-PAGE analysis of recombinant protein expressed in *E. coli* BL21 (DE3) cells. Lane M, protein marker; lane 1, supernatant fraction from cell lysate of bacteria transformed with pGEX6p-2/Cpn0423 with IPTG (1 mM) induction for 4 h; lane 2, PBS Washings; lane 3: Protein Cpn0423 first eluent from glutathione sepharose; lane 4: Purified protein Cpn0423 from glutathione sepharose. **d** Immunoblotting of Cpn0423: eluent buffer (Con.) and purified Cpn0423 were immunoblotted for Cpn0423

The Western blot assay was carried out as described previously [27]. Briefly, Proteins were resolved in SDS-PAGE gels and immunoblotted with mouse pAbs against Cpn0423 (Fig. 1d), NOD2 and GAPDH (Fig. 4c) (Invitrogen) respectively. The primary antibody binding was probed with a horseradishperoxidase-conjugated goat anti-mouse IgG secondary antibody and was visualized with an enhanced chemiluminescence (ECL) kit (Sangon Biotech).

### Cpn0423 in vivo administration

Male wild-type C57BL/6 mice (6–8 weeks old) were purchased from Model Animal Research Centre of Nanjing University. Mice were fed with bacteria-free diet and water. For Cpn0423 in vivo experiments, simple randomization method was used to assign animals to various experimental conditions. All mice used were gender and age matched. Firstly, Cpn0423 was pretreated with PTR according to the manufacture’s instruction. WT mice were then administered with Cpn0423 pretreated with PTR (50 mg/kg), or phosphate-buffered saline (PBS), PTR alone, Cpn0423 (50 mg/kg) alone as controls through intranasal administration respectively. BALF and lung tissue were collected 15 days after Cpn0423 treatment as described [29]. Briefly, 1 ml of PBS free of ionized calcium and magnesium was instilled four times via the tracheal cannula and was recollected by gentle manual aspiration. The fluids from each mouse were combined and immediately centrifuged at 700 *g* for 10 min (4 °C). The BALF supernatants were saved for cytokine measurements, and the cell pellets of BALF were suspended, manually counted and classified. The lung tissues were fixed with 4% paraformaldehyde fixative solution for histopathology examination, and the sections were stained with hematoxylin and eosin (H&E). Inflammation scores in the lungs were performed in a blinded fashion using a reproducible scoring system, as previously described [30].

### Statistical analysis

Statistical analysis was performed using Graphpad Prism 5 software (Graphpad, La Jolla, CA). Data were expressed as the mean ± SE, and statistical analysis of the data was performed using ANOVA and *t* tests, Statistical differences were considered to be significant when *P* < 0.05 for tests with one comparison.

## Results

### Preparation of Cpn0423 fusion protein

Cpn0423 gene was amplified by PCR using *C. pneumoniae* strain AR-39 genomic DNA. The specific primers amplified a 429 bp fragment (Fig. 1a). The digested PCR product (with Bam H I and Not I) was cloned into pGEX6p-2 vector and sequenced (Fig. 1b). The constructed vector, which expressed a soluble protein with the expected molecular mass of 41 kDa, was transformed into *E. coli* BL21cells. The recombinant protein was induced by IPTG and purified by using affinity chromatography (Fig. 1c). And the purified Cpn0423 protein reacted well with Cpn0423 polyclonal antibody (pAb) raised in this study (Fig. 1d). Endotoxin of purified recombinant protein used in this study was also removed by using commercial kits, and the final concentration of endotoxin was less than 60 pg/ml (Data not shown).

### Cpn0423 is detected outside of *C. pneumoniae* inclusions

**Fig. 2:**
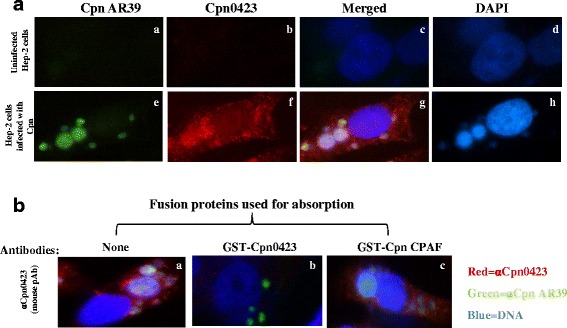
Immunofluorescence detection of Cpn0423 in the *C. pneumoniae*-infected Hep-2 cells. Hep-2 cells infected with *C. pneumoniae* (*A,* panels **a**-**d**) or uninfected Hep-2 cells (*A,* panels **e**-**h**) were stained with mouse anti-Cpn0423 fusion protein antibodies visualized with a goat anti-mouse IgG-conjugated Cy3 (*red*). Cells were co-stained with a rabbit anti-Cpn AR39 visualized with a Cy2-conjugated goat anti-rabbit IgG (*green*) and a DNA Hoechst dye (*blue*). *B,* Cpn0423 pAb was pre-absorbed with Cpn0423and CPAF fusion proteins respectively, followed by immunostaining as described for panel A. The antibody pre-absorption was carried out as previously described [25]. Note that anti-Cpn0423 antibody labeling were removed by pre-absorption with the corresponding (B, panel **b**) but not heterologous CPAF (B, panel **c**) fusion proteins

Mice were immunized with the purified Cpn0423 fusion protein to raise polyclonal antibody (pAb), which were used to determine the localization of the endogenous Cpn0423 protein in *C. pneumoniae*-infected Hep-2 cells through immunofluorescence assay. As shown in Fig. 2a, the vast majority of Cpn0423 were detected in the cytosol of host cell infected with *C. pneumoniae*, but not in those of uninfected host cell, suggesting that Cpn0423 was mainly localized in the host cell cytosol, a pattern similar to CPAF protein (a chlamydial serine protease known to be secreted into the host cell cytosol), but distinct from those of chlamydial inclusion membrane protein [31]. We next confirmed the antibody labeling specificity by using an absorption procedure (Fig. 2b). The granular staining inside inclusion and the diffused staining in the host cell cytosol labeled by the anti-Cpn0423 antiserum was removed by absorption with GST- Cpn0423 but not GST-CPAF fusion proteins, demonstrating that anti-Cpn0423 antibodies specifically labeled the corresponding endogenous proteins without cross-reacting with CPAF. Resolving the precise identification and localization of Cpn0423 will require anti-Cpn0423 monoclonal (mAb) and techniques with higher resolution like confocal microscopy, which will be carried in the future. The above observations have demonstrated that Cpn0423 at least can be detected outside of *C. pneumoniae* inclusions.

### Cpn0423 induces cytokines production in BMDM

**Fig. 3:**
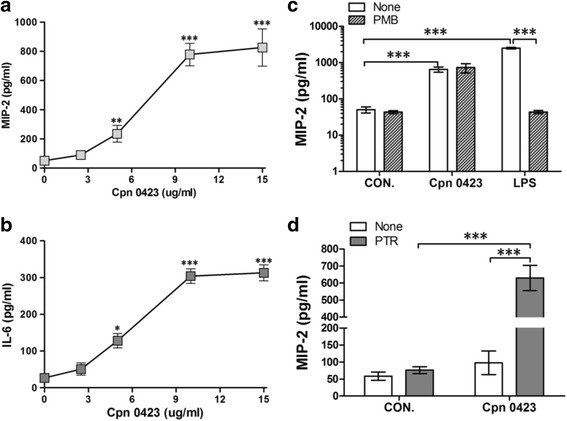
Cpn0423 fusion protein induces cytokine production. Bone marrow-derived macrophages (BMDMs***,*** *A-C*) were treated with Cpn0423 in the presence of PTR. Cells plated in 96-wells were treated for 18-24 h and the media were harvested for cytokines protein measurement. **a** Dose course of Cpn0423-induced MIP-2 production in BMDMs. **b** Dose course of Cpn0423-induced IL-6 production in BMDMs. **c** Effect of polymyxin B sulfate (PMB, a LPS neutralizer) on Cpn0423 MIP-2 production. Cpn0423 (10 μg/ml) or LPS (10 ng/ml) was incubated with 50 μg/ml PMB for 30 min at 4 °C before treatment. **d** Effect of PTR on Cpn0423-induced MIP-2 production. BMDMs were treated with 10 μg/ml Cpn0423 pre-treated with or without PTR (None group). Each error bar represented Mean ± SD. Two-tailed unpaired student *t* test was used in Fig. 3.*A-D*, * *P* < 0.05, ** *P* < 0.01, *** *P* < 0.001. *n* = 3 in each group. PMB: polymyxin B sulfate; PTR: Protein Transfection Reagent

To determine the effect of Cpn0423 on cytokines production, we treated BMDM with purified and endotoxins-removed Cpn0423. As shown in Fig. 3a, Cpn0423 induced an increase of MIP-2 protein production in a dose-dependent (2.5-15 μg/ml) manner at 16 h, whilst it also induced a dose-dependent (2.5-15 μg/ml) increase in IL-6 production at 16 h (Fig. 3b). Since endotoxins have strong biological effects even at very low concentrations in vivo and in vitro, we pretreated Cpn0423 with PMB, a LPS neutralizer, to exclude the interference of any potential endotoxins contamination during Cpn0423 purification or treatment. Results showed in Fig. 3c confirmed that PMB effectively ruled out the possibility of potential LPS contamination without affecting MIP-2 response induced by Cpn0423. Furthermore, Cpn0423-induced cytokine production in BMDM was dependent on pretreatment with PTR, a liposome transfection reagent [32] that entered the cell via either direct fusion with the plasma membrane or by endocytosis and subsequent fusion with the endosome, releasing the captured protein into the cytoplasm (Fig. 3d). In all following experiments, we used 10 μg/ml of Cpn0423 complexed with PTR.

### Cpn0423-induced cytokine response is attenuated by NOD2-siRNA

**Fig. 4:**
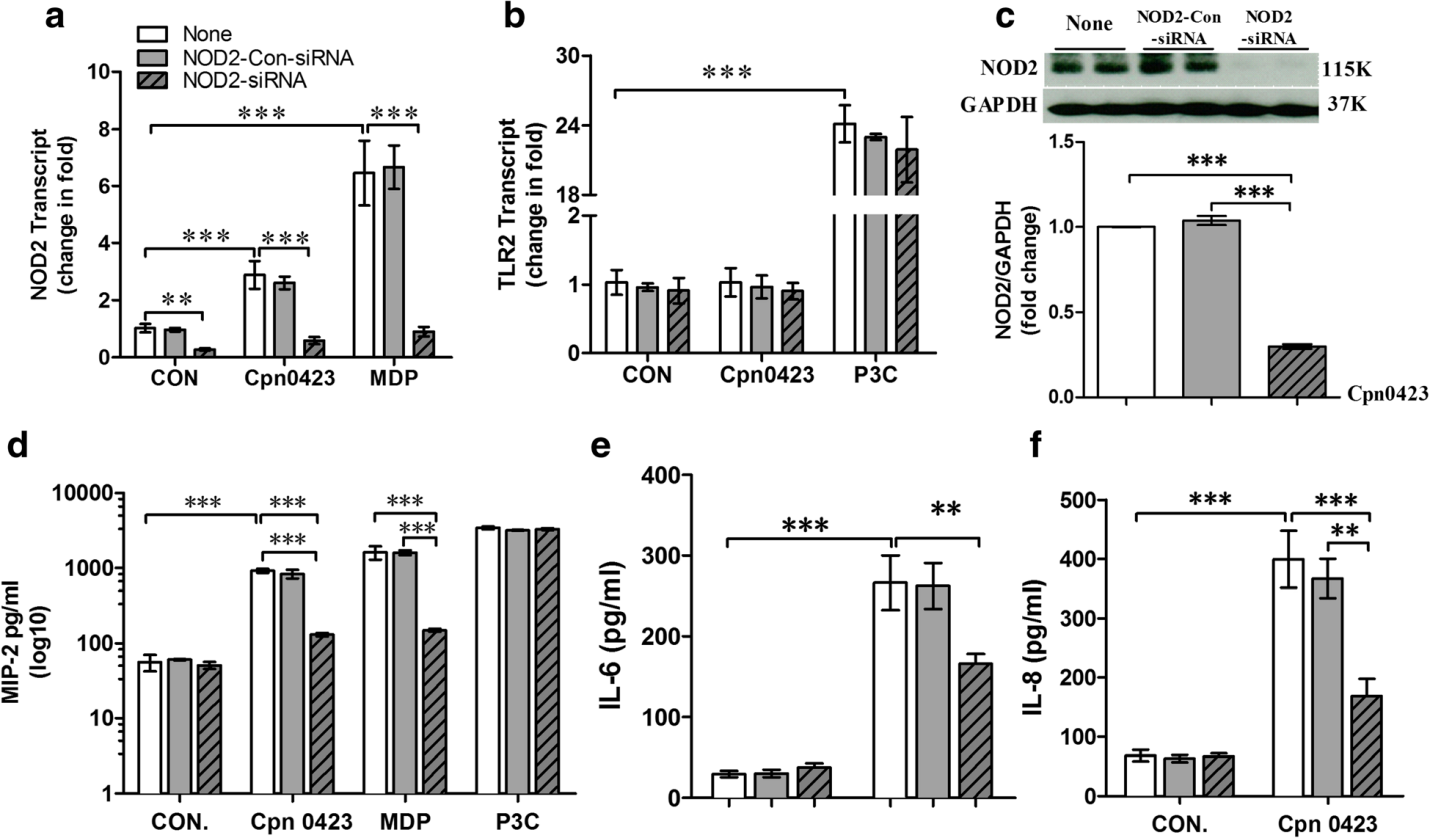
NOD2 mediates Cpn0423-induced cytokine production. Bone marrow-derived macrophages (BMDMs) were pretreated respectively with lipofectamine-complexed NOD2-siRNA or sterile water for 48 h before the administration of Cpn0423 (10 μg/ml), MDP (NOD2 ligand, 20 ng/ml), and Pam3Cys (P3C, TLR2 ligand, 10 ng/ml). mRNA and protein levels of NOD2 or TLR2 were assessed by quantitative real-time PCR and western blot respectively, and expression was normalized to that of GAPDH (**a**-**c**). Sixteen hours later, the culture media were collected and cytokine expression in the media was measured by MIP-2, IL-6 and TNF-α ELISA respectively (**d**-**f**). Each error bar represented mean ± SD. Two-tailed unpaired student *t* test was used in Fig. 4. **a**-**e** * *P* < 0.05, ** *P* < 0.01, *** *P* < 0.001. *n* = 3 in each group

The cytosolic pattern recognition receptor NOD2 can be activated to generate a proinflammatory immune responses, and the early immune response involves the influx of macrophages [6]. We hypothesized that NOD2 signaling was responsible for the Cpn0423-induced cytokine production. To test the hypothesis, we synthesized the specific NOD2-siRNA and NOD2-Con-siRNA oligonucleotides. We performed quantitative PCR and western blot to determine the mRNA and protein expression levels of NOD2 receptor in BMDM. As shown in Fig. 4a, b and c, compared to the NOD2-Con-siRNA oligonucleotide sequence, NOD2-siRNA pretreatment resulted in significant, but incomplete, reduction of NOD2. NOD2-siRNA also reduced NOD2 receptor mRNA level induced by MDP and attenuated NOD2 protein expression induced by Cpn0423, respectively. However, the mRNA level of TLR2 receptor expression induced by P3C, a TLR2 ligand, was unaffected. Of note, Cpn0423 induced NOD2, but not TLR2, the expression in BMDM, and the specificity of NOD2-siRNA was confirmed by its selective inhibitory effect on MDP-induced mRNA expression of NOD2.

To further test the impact of NOD2 signaling on Cpn0423-induced cytokine responses, we treated BMDM with Cpn0423, NOD2 and TLR2 ligand. As anticipated, Cpn0423 and NOD2 induced robust MIP-2 protein production in BMDMs (Fig. 4d). However, MIP-2 protein production was lessened significantly but incompletely by about 89.7% and 85.6%, respectively, after NOD2-siRNA treatment, while P3C-induced MIP-2 protein production remained unaffected. We also tested other cytokines productions in responses to Cpn0423. Similarly, Cpn0423-induced IL-6 and TNF-α productions were significantly decreased in BMDMs pretreated with NOD2-siRNA, but remained the same after NOD2-Con-siRNA pretreatment (Fig. 4e and f).

### Intranasal Cpn0423 induces pulmonary inflammation in vivo

**Fig. 5:**
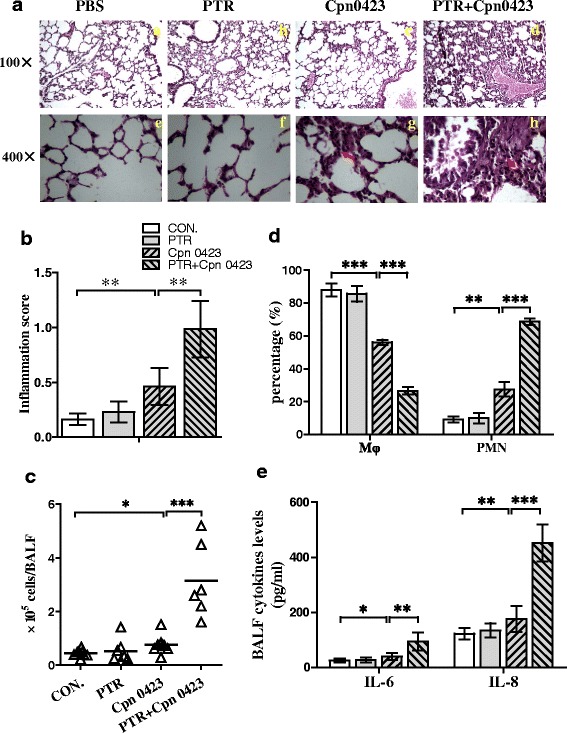
Effects of Cpn0423 on pulmonary inflammation in vivo*. A,* Histopathological analysis of lung tissues from mice of all the experimental groups were performed at days 15 after intranasal administration (haematoxylin and eosin staining, panels *a-d* magnification 100×; panels **e**-**h**: magnification 400×). Mice were treated with PBS (**a** & **e**), PTR (**b** & **f**), Cpn0423 (50 mg/kg) (**c** & **g**), and PTR + Cpn0423 (50 mg/kg) (**d** & **h**). *B,* Results of histological scoring for pulmonary inflammatory changes of lung sections from different groups of mice. A score of 0–3 was adjudged to the tissue sections of the mice as described in Materials and methods. *C,* The total BALF cells in each group. *D,* The percentage of macrophages (Mϕ) and polymorphonuclear leukocyte (PMN) among total BALF cells. *E,* Levels of the cytokines IL-6 and TNF-α in BALF were measured using enzyme-linked immunosorbent assay. Each error bar represented mean ± SD. Two-tailed unpaired student *t* test was used in Fig. 5. *E-H*, ** *P* < 0.01, *** *P* < 0.001. *n* = 8 mice in PBS Group, *n* = 7 mice in PTR-control Group, *n* = 8 mice in Cpn0423 group, *n* = 6 mice in Cpn0423 + PTR group. PTR: Protein Transfection Reagent. Red arrowhead indicates inflammatory cells infiltration

To evaluate the ability of Cpn0423 to induce pulmonary inflammation in vivo, mice were treated with Cpn0423 pretreated with PTR, Cpn0423 alone, PBS and PTR alone via intranasal administration respectively. Histopathology analysis of lung sections 15 days after administration showed there was no histological evidence of inflammation in PBS and PTR control group mice, but detectable inflammation could be observed in Cpn0423 and Cpn0423 pretreated with PTR group mice (Fig. 5a). As shown in Fig. 5b, Cpn0423 significantly increased inflammatory scores significantly compared to PBS and PTR control group (*P* < 0.01), which was markedly enhanced by Cpn0423 pre-treatment with PTR (Fig. 5b), suggesting an essential role of PTR on Cpn0423-induced inflammatory response.

To investigate the effect of Cpn0423 on leukocyte recruitment, we calculated the total cell number of BALF in mice as presented in Fig. 5c. Evaluation showed no significant differences in total cell number in PBS (0.43 ± 0.13 × 10^5^) and PTR group (0.50 ± 0.43 × 10^5^) (*P > 0.05*), while total cell increase in BALF was observed in Cpn0423 group (0.76 ± 0.35 × 10^*5*^), and the cell number increase to 2.32 ± 0.42 × 10^5^ after PTR pre-treatment (*P < 0.01*). BALF cell classification revealed a decrease in macrophages (88 ± 4.02% vs. 56.18% ± 4.04%, *P < 0.001*) and an increase in polymorphonuclear leukocyte (PMN) (9.18 ± 2.79% vs. 27.56 ± 12.61%, *P < 0.01*) in the Cpn0423 administered mice compared to the control mice with PBS treatment. Similarly, PTR pre-treatment significantly improved the role of Cpn0423 on macrophages (26.58% ± 5.62%, *P < 0.001*) and PMN (68.75% ± 4.71%, *P < 0.001*) cell classification (Fig. 5d), suggesting that Cpn0423 induced inflammatory cells recruitment and affected the ratio of macrophages and PMN. To gain insight into the effect of Cpn0423 on inflammation, we detected the changes of IL-6 and TNF-α in the BALF supernatant. As expected, intranasal administration of Cpn0423 or Cpn0423 pre-treated with PTR both resulted in a significant increase in IL-6 (*P < 0.05* & *P < 0.01*) and TNF-α (*P < 0.01* & *P < 0.001*) levels in the BALF (Fig. 5e). These data indicated that Cpn0423 induced pulmonary inflammation in vivo, consistent with our in vitro observations.

## Discussion

Chronic *C. pneumoniae* infections have been associated with the induction or the acceleration of various inflammatory diseases [33]. Although a few studies have demonstrated that *C. pneumoniae* heat shock protein 60 (HSP60) activates NF-κB in a MyD88-independent TLR4 manner [34], the cellular and molecular mechanisms by which *C. pneumoniae* participates in the inflammatory responses remain to be elucidated. Hence it is important to identify the exact components of *C. pneumoniae* and the pattern recognition receptors which initiate or are involved in the inflammatory responses. In the current study, we made three main findings. First, we found that Cpn0423 was detected in the cytosol of *C. pneumoniae*-infected cells, and we detected robust cytokine responses in BMDMs when they were treated with Cpn0423. Second, NOD2 signaling was involved in the Cpn0423-induced MIP-2 expression. Third, Cpn0423 intranasal administration in vivo induced pulmonary inflammation and caused pneumonia.

*C. pneumoniae* are obligate intracellular parasites that only live inside the host cells to ensure their successful survival, which has been shown to infect and productively replicate within a number of cell types [5], including macrophages. Therefore, the components of *C. pneumoniae* might fulfill its function mostly inside the host cells. Considering that, we transfected Cpn0423 into BMDMs using lipid-based PTR, and our data demonstrated that the cytokine-producing effect of Cpn0423 was markedly enhanced and dependent on the presence of PTR.

Endotoxins liberated by gram-negative bacteria are frequent contaminations of protein solutions derived from bioprocesses and showed strong biological effects at very low concentrations [35]. Therefore, their removal is essential for recombinant proteins administration. Herein, we conducted the removal of LPS from Cpn0423 by Endotoxin Removal Resin. Our data showed that PMB pretreatment totally diminished LPS-induced MIP-2; in contrast, it did not affect Cpn0423-induced MIP-2 response, suggesting efficient endotoxin removal from Cpn0423 in our study.

Innate immunity is an evolutionarily ancient part, which lies behind most inflammatory responses. Innate immune recognition relies on a limited number of germline-encoded receptors, including cell surface receptors, TLR2, TLR4, and TLR11, and intracellular pattern recognition receptors, e.g. NOD2, TLR3, and TLR9 [13, 15]. Recent studies strongly support an important role of *C. pneumoniae* HSPs, LPS and MOMP played in driving innate immune responses [16, 36], while TLR2 and TLR4 are both important to orchestrate cytokine and chemokine responses and host defense against *C. pneumoniae* infection in vivo. TLR3 and TLR9 are located primarily on the membranes of intracellular compartments and mainly responsible for the recognition of microbial nucleic acids [13]. NOD2 is a kind of cytosolic proteins that respond to intracellular fragments of bacterial peptidoglycan, which is also crucial for innate immune responses to certain bacterial infections [13, 20]. *C. pneumoniae* can gain access to the intracellular compartments, whereas the link between *C. pneumoniae* and NOD2 has been barely studied. In this study, Cpn0423 was detected in the cytosol of *C. pneumoniae*-infected cells, and the detection of the endogenous Cpn0423 was specific. The anti-Cpn0423 antibody labeling observed under the fluorescence microscope was only removed by absorption with Cpn0423 but not CPAF fusion protein. Moreover, we characterized NOD2 expression in BMDMs as well as the specificity of NOD2-siRNA, and found that NOD2-siRNA pretreatment dramatically attenuated Cpn0423-induced cytokine responses in BMDMs, suggesting that NOD2 was involved in Cpn0423-induced cellular inflammation. Although our results suggest a role for NOD2 in Cpn0423-induced inflammatory response, the exact downstream signaling cascade remains to be elucidated. One candidate molecule is the receptor interacting protein kinase 2 [37], which has been suggested to link NOD2 and efficient immune responses during *C. pneumoniae* infection.

*C. pneumoniae* infection might contribute to chronic inflammatory events associated with pneumonia [38], whereas, the exact components of *C. pneumoniae* which activate immune system in vivo have been barely studied. In the present study, we have presented convincing evidence that *C. pneumoniae* protein Cpn0423 could cause pneumonia and induce a strong immune response. After Cpn0423 intranasal administration, we observed increased infiltrating inflammatory cells in the lung histology, recruitment of a large number of immune cells with a higher percentage of neutrophils and lower percentage of macrophages, and higher cytokines levels in the BALF. Macrophages, as resident cells of almost every tissue in the body, may be stimulated first by Cpn0423, and secreted chemokines such as MIP-2 and IL-8 to trigger neutrophils accumulation in a cascade amplification way [39, 40]. This, may explain the proportion changes of neutrophils and macrophages. Interestingly, unlike in vitro, inflammatory response was also found in mice administered with Cpn0423 alone, the possible reason maybe some compounds in the body could help Cpn0423 enter into the host cells to fulfil its function.

We detected marked cytokine responses both in vitro and in vivo upon treatment with Cpn0423. Whether the dose of Cpn0423 used in this study corresponded to the one generated during a natural infection remains unknown. However, *C. pneumonia* infection has a gradual onset, and the organism often reside intracellularly and secrete various proteins with different quantities for indefinite periods, potentially promoting chronic inflammation [4, 41]. To the best of our knowledge, it is difficult to evaluate how much *C. pneumonia* protein can be secreted from infected host cells or what *C. pneumonia* protein amount are presented in infected cells. Thus, the concentration of Cpn0423 used in this paper was based on our pilot studies and previous reported [16, 34]. Here we showed for the first time the discovery of recombinant protein Cpn0423 as an activator and its effects on pneumonia. We will continue to clarify the mechanism of entry and presentation of Cpn0423 to NOD2, the precise NOD2 downstream molecular signaling in Cpn0423-induced inflammation, and the role of endogenous Cpn0423 during *C. pneumoniae* infection.

## Conclusions

In the present paper, we demonstrated that *C. pneumoniae* protein could act as signals for intracellular pattern recognition receptors activation. Specially, we found that Cpn0423 was an activator of NOD2 and provoked profound inflammatory responses in BMDMs. We also identified the pivotal role of Cpn0423 in mediating pulmonary inflammation in vivo*.* We speculated that Cpn0423 exists under during *C. pneumoniae* infection conditons and stimulated NOD2, thereby accelerating host immune responses against *C. pneumoniae.* These observations suggest that Cpn0423 is a potent and specific innate immune activator that may initiate a local inflammation by inducing cytokine release. Further characterization of the inflammatory effect of *C. pneumoniae* protein will speed up our understanding of the pathogenic mechanism of *C. pneumoniae*.

## Abbreviations

BALF: Bronchoalveolar lavage fluid
BMDM: bone marrow-derived macrophages
Cpn: *Chlamydia pneumoniae*
*IL*: *interleukin*
MDP: muramyl dipeptide
MIP-2: macrophage inflammatory protein-2
NOD2: Nucleotide-binding oligomerization domain-containing protein 2
NOD2-Con-siRNA: small interfering RNA negative control for NOD2
NOD2-siRNA: small interfering RNA against NOD2
P3C: Pam_3_Cys-Ser-(Lys)_4_
TLR: Toll-like receptors
